# The use of chemotherapy regimens carrying a moderate or high risk of febrile neutropenia and the corresponding management of febrile neutropenia: an expert survey in breast cancer and non-Hodgkin's lymphoma

**DOI:** 10.1186/1471-2407-10-642

**Published:** 2010-11-23

**Authors:** Laetitia Gerlier, Mark Lamotte, Ahmad Awada, André Bosly, Greet Bries, Véronique Cocquyt, Christian Focan, Stéphanie Henry, Yassine Lalami, Jean-Pascal Machiels, Jeroen Mebis, Nicole Straetmans, Didier Verhoeven, Luc Somers

**Affiliations:** 1 Health Economics and Outcomes Research Department, IMS Health Consulting, Medialaan 38, 1800 Vilvoorde, Belgium; 2 Medical Oncology Clinic, Jules Bordet Institute, boulevard de Waterloo, 121, B-1000 Brussels, Belgium; 3 Department of Hematology, University Hospital of Mont-Godinne, Avenue Dr G. Therasse, 1, B-5530 Yvoir, Belgium; 4 Department of Hematology, Virga Jesse Hospital, Stadsomvaart, 11, B-3500 Hasselt, Belgium; 5 Medical Oncology, University Hospital Ghent, De Pintelaan, 185, B-9000 Gent, Belgium; 6 Department of Oncology, CHC-Saint-Joseph Clinic, rue de Hesbaye, 75, B-4000 Liège, Belgium; 7 Department of Oncology, University Hospital of Mont-Godinne, Avenue Dr G. Therasse, 1, B-5530 Yvoir, Belgium; 8 Medical Oncology, Sainte-Elisabeth Clinic, place Louise Godin, 15, B-5000 Namur, Belgium; 9 Medical Oncology, UCL Saint-Luc University Hospital, Avenue Hippocrate 10, B-1200 Brussels, Belgium; 10 Medical Oncology, Virga Jesse Hospital, Stadsomvaart, 11, B-3500 Hasselt, Belgium; 11 Department of Hematology, Jolimont Hospital, rue Ferrer, 159, B-7100 Haine-Saint-Paul, Belgium; 12 Medical Oncology, Iridiumkankernetwerk, AZ Klina, Augustijnslei 100, B-2930 Brasschaat, Belgium; 13 OncoLogX, Arthur Boelstraat 66, B-2990 Wuustwezel, Belgium

## Abstract

**Background:**

The use of chemotherapy regimens with moderate or high risk of febrile neutropenia (defined as having a FN incidence of 10% or more) and the respective incidence and clinical management of FN in breast cancer and NHL has not been studied in Belgium. The existence of a medical need for G-CSF primary and secondary prophylaxis with these regimens was investigated in a real-life setting.

**Methods:**

Nine oncologists and six hematologists from different Belgian general hospitals and university centers were surveyed to collect expert opinion and real-life data (year 2007) on the use of chemotherapy regimens with moderate or high risk of febrile neutropenia and the clinical management of FN in patients aged <65 years with breast cancer or NHL. Data were retrospectively obtained, over a 6-month observation period.

**Results:**

The most frequently used regimens in breast cancer patients (n = 161) were FEC (45%), FEC-T (37%) and docetaxel alone (6%). In NHL patients (n = 39), R-CHOP-21 (33%) and R-ACVBP-14 (15%) were mainly used. Without G-CSF primary prophylaxis (PP), FN occurred in 31% of breast cancer patients, and 13% had PSN. After G-CSF secondary prophylaxis (SP), 4% experienced further FN events. Only 1 breast cancer patient received PP, and did not experience a severe neutropenic event. Overall, 30% of chemotherapy cycles observed in breast cancer patients were protected by PP/SP. In 10 NHL patients receiving PP, 2 (20%) developed FN, whereas 13 (45%) of the 29 patients without PP developed FN and 3 (10%) PSN. Overall, 55% of chemotherapy cycles observed in NHL patients were protected by PP/SP. Impaired chemotherapy delivery (timing and/or dose) was reported in 40% (breast cancer) and 38% (NHL) of patients developing FN. Based on oncologist expert opinion, hospitalization rates for FN (average length of stay) without and with PP were, respectively, 48% (4.2 days) and 19% (1.5 days). Similar rates were obtained from hematologists.

**Conclusions:**

Despite the studied chemotherapy regimens being known to be associated with a moderate or high risk of FN, upfront G-CSF prophylaxis was rarely used. The observed incidence of severe neutropenic events without G-CSF prophylaxis was higher than generally reported in the literature. The impact on medical resources used is sizeable.

## Background

Severe neutropenic events are life-threatening conditions that can lead to serious infection, long-lasting hospitalization and death, on top of being a dose-limiting toxicity of cancer chemotherapy. A retrospective analysis of a 1999 hospital discharge database, which included data from seven states in the USA, reported a mortality rate of 6.8% for patients hospitalized for FN, equating to one death for every 14 hospitalized patients [1]. In another study, the reported overall in-hospital mortality with FN was 9.5% [2], while a recent claims database study showed a significant increase in the risk of overall and early mortality in patients with FN compared to controls by 15% and 35% respectively [3]. Moreover, severe neutropenia often leads to a delay or dose reduction in planned chemotherapy [4,5] and has been shown to negatively influence patient quality of life [6,7].

Several randomized controlled trials demonstrated a significant reduction in FN after systemic chemotherapy with the use of prophylactic G-CSF compared with untreated controls, and a recent meta-analysis on the proactive use of G-CSF as primary prophylaxis revealed a significant reduction in the risk of FN (RR = 0.54) and infection-related mortality (RR = 0.55) with this strategy, together with a significant increase in the relative dose intensity of the chemotherapy administered (+8.4% on average) [8]. A US claims database study confirmed the use of G-CSF prophylaxis to significantly reduce the hazard of overall mortality (HR = 0.65 [0.53; 0.79]) [3]. Conversely, the absence of G-CSF prophylaxis was significantly associated with higher rate of FN and reduced relative dose-intensity in NHL patients from two retrospective studies [9,10]. Other studies, in both clinical trial and community settings, have demonstrated that FN events mainly occur during the first cycles of chemotherapy, thereby highlighting the importance of primary prophylaxis in patients at high risk for FN [11-14].

International guidelines concerning the use of G-CSF have been issued by professional cancer associations such as ASCO, EORTC, ESMO and NCCN [15-17].

According to these guidelines, a patient's overall risk for FN is comprised of two components: the type of chemotherapy itself and patient-related factors such as age, performance status and comorbidities. Taking these factors into account, patients can be allocated to one of three FN risk groups: low risk (< 10%), intermediate risk (10-20%) and high-risk (≥20%). All guidelines make similar recommendations regarding the use of G-CSF as primary prophylaxis in patients with an overall high risk of FN (e.g. ≥ 20%).

Both the EORTC and ASCO guidelines present good evidence that prophylactic G-CSF decreases the incidence of dose reductions and delays, and advocate the use of primary prophylaxis to maintain the intended dose intensity of chemotherapy when survival benefits are expected, such as in patients with breast cancer or NHL.

Although clinical studies have demonstrated the risk of FN for several chemotherapy regimens in a clinical study setting, few attempts have been made to describe the occurrence of FN in real-life settings beyond the strict context of a clinical trial [18,19]. Likewise, although international guidelines are clear on the use of G-CSF prophylaxis, data on the use of G-CSF for FN prophylaxis in real-life practice are scarce.

In Belgium, access to a specific therapy is largely dependent on the reimbursement criteria centrally regulated by the Social Security Authority (RIZIV-INAMI), and no data have been published on the actual use of G-CSF in different patient settings in Belgium. During the period of our study, patients with breast cancer or NHL younger than 65 years of age were eligible for the reimbursement of G-CSF as prophylaxis only when used as secondary prevention ('reactive' use). It is therefore expected that the use of G-CSF for primary prevention will be rather limited in these patients. The primary objective of this survey was therefore to obtain real-life data on the use of chemotherapy regimens documented in clinical trials to provide a moderate or high risk of FN (incidence >= 10%), including the observed incidence of FN in daily practice. A secondary objective was to obtain expert opinion on the current management of FN in a Belgian clinical setting. It was anticipated that the survey outcome would contribute to a re-evaluation of the medical need for G-CSF prophylaxis with the use of these regimens in these specific types of patients, and provide basic data with which to analyze the budgetary implications of a potential enlargement of the Belgian reimbursement criteria.

## Methods

The study was designed as a written expert survey. Two similar but separate surveys were conducted, one involving a panel of oncologists (indication breast cancer) and one involving hematologists (indication NHL). Experts from both general hospitals and university centers were included.

Two documents were completed by each investigator. The first document comprised a spreadsheet for the collection of retrospective individual patient data from patient files or departmental databases. This patient-level information was automatically summarized per chemotherapy regimen on a separate spreadsheet, providing aggregated numbers only. The second document comprised a written questionnaire which asked the investigator about his/her daily practice relating to G-CSF prophylactic use and FN management (and hence was not based on real patient data).

### Definitions

Definitions of primary and secondary G-CSF prophylaxis were provided in the survey protocol sent to all participating centers. Primary G-CSF prophylaxis (PP) was defined as the administration of G-CSF from the first cycle of chemotherapy and for subsequent cycles. Two types of secondary prophylaxis (SP) were distinguished, driven by the first neutropenic event: administration of G-CSF in the cycles following the occurrence of FN (SP-FN) and administration of G-CSF following the occurrence of PSN (SP-PSN) during a chemotherapy cycle. In the current paper, a severe neutropenic event refers to either FN or PSN (defined as an episode of severe neutropenia grade 4, without fever, for ≥ 5 days).

### Real patient-based information: patient selection

Data on patients aged < 65 years and treated with a cytotoxic chemotherapy regimen for breast cancer or aggressive NHL in the investigator's department between 01 April 2007 and 30 September 2007 (recruitment period) were included. Each investigator enrolled a maximum of 25 breast cancer or 15 NHL patients, and included the most recently and consecutively treated patients. The FN risk of chemotherapy regimens was assessed according to EORTC guidelines, by type of malignancy.

For each eligible patient, the observation period started on the first cycle of the chemotherapy regimen administered at recruitment and stopped after 6 months, or earlier in case of death, loss-to-follow-up or if switched to another protocol. This duration of 6 months was chosen in order to capture at least 6-8 chemotherapy cycles of 28, 21 or 14 days' duration.

### Real patient-based information: variables collected

The investigator was asked to provide the following information per patient: age, prior FN (yes/no), prior/concomitant radiotherapy (yes/no), use of G-CSF prophylaxis for FN (yes/no), name of the chemotherapy regimen received, number of cycles observed, occurrence of FN during the observation period (yes/no) and consequences of FN on the chemotherapy (dose adjustment, delay, cycles dropped, switch to another regimen).

Information collected that was specific to breast cancer included: disease stage (early/locally advanced or metastatic), presence of bone metastasis (yes/no) and prior/concomitant surgery or hormone therapy (yes/no). Information specific to NHL included: International Prognosis Index (IPI) and the Ann Arbor classification [20,21].

### Information retrieved as expert opinion

Variables collected in the expert survey included center location, number of beds, usage of chemotherapy regimens with moderate or high risk of FN (from a predetermined list of regimens or possibly institution-based regimens), G-CSF primary or secondary prophylaxis (proportional use by drug, dosage, duration) and clinical management of FN (type and frequency of medications, procedures, hospitalizations, length of stay with or without G-CSF prophylaxis).

### Statistical analysis

The analysis was descriptive. The primary objective of the study was to estimate the proportional use of chemotherapy regimens carrying a moderate or high risk of FN in patients with breast cancer or NHL.

As part of the secondary objectives, the study sample was described in terms of real-life occurrence of neutropenic events (FN/PSN), presence of G-CSF prophylaxis and consequences of FN on the chemotherapy protocol (percentage of patients who experienced dose adjustment or delay, dropped cycles or were switched to another regimen as a result of developing FN). Results were presented by indication and by regimen subgroups where sample size allowed.

Finally, information from experts on the type of G-CSF used and the clinical management of FN was summarized using descriptive statistics, based on the average of investigators' answers.

## Results

In total, nine oncologists and six hematologists spread regionally (eight from the south and five from the north of Belgium, two from Brussels) and representing university (seven) and general (eight) hospitals contributed data to both parts of the survey. The participation rate was 45% in both indications. Real-life data were collected for 161 breast cancer (969 cycles of chemotherapy observed) and 39 NHL patients (208 cycles).

### Breast cancer patients - characteristics and proportional use of chemotherapy regimens

The 161 breast cancer patients were aged between 21 and 65 years; 88% had early/locally advanced disease and 12% had metastatic disease (7% with bone metastasis). A majority of women had already been treated with surgery (90%), radiotherapy (69%) and/or hormone therapy (56%). Only two patients (1%) had experienced a prior FN episode.

The three most frequently used regimens associated with a moderate or high risk of FN used in breast cancer were: FEC (45%), FEC-T (3 or 4 cycles each, 37%) and docetaxel alone (6%). Other regimens with a moderate risk of FN were used in only one or two breast cancer patients (Table 1).

**Table 1:** Basic characteristics of breast cancer patients from the study sample

Basic demographic & clinical characteristics	FEC (n = 73)	FEC-T (n = 60)	**All regimens (n = 161) **^**a**^
Age range (years)	28-63	27-65	21-65

Disease stage, n (%) metastatic	12 (16)	1 (2)	20 (12)

Bone metastasis, n (%)	7 (10)	0 (0)	12 (7)

HER status, n (%) positive	17 (23)	25 (42)	53 (33)

Prior/concomitant surgery, n (%)	67 (92)	53 (88)	145 (90)

Prior/concomitant radiotherapy, n (%)	49 (67)	40 (67)	111 (69)

Prior/concomitant hormone therapy, n (%)	42 (58)	33 (55)	90 (56)

Basic demographic and clinical characteristics of the breast cancer patients are presented for the two most frequent chemotherapy regimens, and all regimens.

^**a**^Other regimens used in the sample but not displayed were docetaxel (n = 9), AC → T (n = 5), capecitabine/docetaxel (n = 2), capecitabine/docetaxel/trastuzumab (n = 2), docetaxel/trastuzumab (n = 2), epirubicin/cyclophosphamide (n = 2), FEC/trastuzumab (n = 2) and capecitabine/larotaxel, doxorubicin/docetaxel, epirubicin/docetaxel, epirubicin/cyclophosphamide/larotaxel (1 patient each)

### Use of G-CSF in FN prophylaxis (breast cancer patients)

Only 1 of the 161 patients with breast cancer (< 1%) received PP, whereas 67 (42%) patients received SP (SP-PSN in 12% and SP-FN in 29%) (Table 2). In total, 30% of the 969 observed chemotherapy cycles were supported by G-CSF prophylaxis. The mean cycle number on which SP was started was 2.1 following PSN and 3.4 following FN, meaning that a first severe neutropenic event occurred mainly in cycle 1 or cycle 2 (Table 2). Of note, three patients with breast cancer developed FN during the last chemotherapy cycle and therefore did not receive SP-FN (see in Table 2, 50 patients developing FN, and only 47 patients receiving SP-FN).

**Table 2:** Description of G-CSF use and occurrence of neutropenic events in study sample (breast cancer and NHL patients)

	Breast cancer regimens			NHL regimens	
**Outcome**	**FEC ** **(n = 73)**	**FEC-T ** **(n = 60)**	**All ** **(n = 161)**	**Dose-dense** ** (n = 11)**	**All** ** (n = 39)**

**Patients with G-CSF use**	n (%)	n (%)	n (%)	n (%)	n (%)

Primary prophylaxis (PP)	0	1 (2)	1 (<1)	3 (27)	10 (26)

Secondary prophylaxis (SP)	36 (49)	23 (38)	67 (42)	7 (64)	16 (41)

due to FN (SP-FN)	23 (32)	17 (28)	47 (29)	6 (55)	13 (33)

**Total patients developing FN**^**a**^	23 (32)	19 (32)	50 (31)	7 (64)	16 (41)

**Cycle # to start SP**^**b**^	Mean cycle #	Mean cycle #	Mean cycle #	Mean cycle #	Mean cycle #

After FN	3.2	3.4	3.4	2.5	2.7

After PSN	2.1	2	2.1	2.0	2.0

**Total number of cycles observed**	424	402	969	49	208

Cycles protected by G-CSF (% observed cycles)	145 (34)	103 (26)	287 (30)	36 (73)	114 (55)

	**Neutropenic events by presence of PP/SP**				

**N patients with at least one unprotected cycle**	73	59	160	8	29

N (%) patients with event in unprotected cycles (first event only)	36 (49)	25 (42)	70 (44)	7 (88)	17 (58)

Of which FN	23 (32)	19 (32)	50 (31)	6 (75)	14 (48)

**N patients with at least one protected cycle**	36	24	68	10	26

N (%) patients with event in protected cycles	2 (6)	0	3 (4)	1 (10)	8 (31)

after PP	NA	0	0	1 (10)	2 (8)

after SP	2 (6)	0	3 (4)	0	6 (23)

	**Impact of FN on chemotherapy delivery (N patients and % among those who developed FN)**				

Dose adjustment	4 (17)	4 (21)	12 (24)	0	0

Dose delay	5 (22)	3 (16)	12 (24)	3 (43)	6 (38)

At least 1 cycle dropped	2 (9)	5 (26)	8 (16)	0	0

Switch chemotherapy protocol	3 (13)	2 (11)	6 (12)	0	0

Any type	8 (35)	8 (42)	20 (40)	3 (43)	6 (38)

This table summarizes, for different subgroups of chemotherapy regimens, the number of patients receiving each type of G-CSF prophylaxis, and the number of patients experiencing severe neutropenic events, depending on the presence of protected/unprotected cycles. The consequences of FN on the chemotherapy delivery are also presented.

^a^Patients who developed FN at the last cycle did not receive SP-FN

^b^If FN in cycle n, SP starts in cycle (n + 1)

NA: not applicable

### Real-life occurrence of neutropenic events (breast cancer patients)

In the 160 patients not receiving PP, 44% developed a severe neutropenic event (50 patients [31%] developed FN and 20 [13%] PSN) - 49% of those receiving FEC, 42% receiving FEC-T and 33% receiving docetaxel. Only three patients had further episodes of FN (4% of 68 patients with cycles protected) after SP was implemented (Table 2).

### Consequences of FN on treatment of breast cancer patients

The consequences of an FN episode in the 50 patients experiencing FN were dose adjustment in 12 breast cancer patients (24% of patients who developed FN), dose delays in 12 patients (24%), cycle dropped in 8 patients (16%) and a switch to another regimen in 6 patients (12%) (Table 2). Overall, 20 (40%) patients developing FN experienced at least one of the above-mentioned impairments to their treatments, of whom 17 were treated with the three most frequently used regimens.

### NHL patients characteristics and proportional use of chemotherapy regimens

Thirty-nine NHL patients aged between 18 and 64 years were analyzed. A majority showed the involvement of one or more extralymphatic organs (62% with Ann Arbor stage IV). IPI was low in 28%, low/intermediate in 36%, high/intermediate in 15% and high in 21% of patients. No prior FN was reported. Prior/concomitant radiotherapy was received by 5% of patients (see Table 3).

**Table 3:** Basic characteristics of patients with NHL from the study sample

Basic demographic & clinical characteristics	3-weekly regimens **(n=28)**^**a**^	Dose dense regimens **(n=11)**^**b**^	All regimens (n=39)
Age range (years)	18-64	20-61	18-64

IPI low, n (%)	7 (25)	4 (36)	11 (28)

IPI low/intermediate, n (%)	11 (39)	3 (27)	14 (36)

IPI high/intermediate, n (%)	5 (18)	1 (9)	6 (15)

IPI high, n (%)	5 (18)	3 (27)	8 (21)

Ann Arbor stade III/IV, n (%)	21 (75)	8 (73)	29 (74)

Prior/concomitant radiotherapy, n (%)	2 (7)	0	2 (5)

Basic demographic and clinical characteristics of the patients with NHL are presented for all 3-weekly chemotherapy regimens, for dose-dense regimens (R-ACVBP-14, ACVBP, R-CHOP-14 and R-etoposide, cyclophosphamide), and for all regimens.

^a ^3-weekly regimens were R-CHOP-21 (n = 13), CHOP-21 (n = 3), BVAM (n = 2), R-DHAP (n = 2), R-GEMOX (n = 2), R-COPADEM (n = 2) and Hyper CVAD, ESHAP, methotrexate/vincristine/ifosfamide/dexamethasone, R-ICE (1 patient each).

^b ^Dose-dense regimens were R-ACVBP-14 (n = 6), ACVBP (n = 2), R-CHOP-14 (n = 2) and R-etoposide-cyclophosphamide (n = 1)

A wide range of regimens with moderate or high risk of FN was used to treat aggressive NHL. The most frequently used regimens in our sample were R-CHOP-21 (in 33% of patients), R-ACVBP-14 (15%) and CHOP-21 (8%).

### Use of G-CSF in FN prophylaxis (NHL patients)

G-CSF was administered to 10 (26%) patients as PP and to 16 (41%) as SP (8% after PSN and 33% after FN). The mean cycle number at which SP was started was 2.0 following PSN and 2.7 following FN, meaning that major neutropenic events occurred mainly during the first chemotherapy cycle (see Table 2). One patient developed a FN episode in the last chemotherapy cycle and therefore did not receive SP-FN. Of 11 patients with NHL treated with a dose-dense chemotherapy only three (27%) received PP.

### Real-life occurrence of neutropenic events in NHL patients

Overall, FN occurred in 16 patients (41%) and PSN in another 3 patients (8%). Among the 10 patients receiving PP, 2 (20%) developed FN. Among the 29 patients not receiving PP, 14 (48%) developed FN and another 3 (10%) experienced PSN; 16 patients (41%) subsequently received SP.

In the 26 patients with at least 1 protected cycle, 8 (31%) experienced at least one new episode of FN in the further course of their treatment (Table 2). Among the 11 NHL patients receiving dose-dense chemotherapy, 3 patients (27%) received PP and 7 (64%) received SP. The rate of FN occurring in protected cycles was 10% (1 FN in a patient receiving PP), while the FN rate in unprotected cycles was 75% (6 FN in the 8 patients with at least 1 unprotected cycle).

### Consequences of FN on treatment of NHL patients

Among the 16 patients who developed FN, 6 (38%) experienced a delay in at least one chemotherapy cycle, but there were no dose adjustments, dropped cycles or switches due to FN (Table 2).

### Expert opinion on prophylaxis and clinical management of FN

On average, the oncologists stated that pegfilgrastim is the drug they most frequently prescribe for PP (to 78% of patients), followed by filgrastim (15%) and lenograstim (7%). Pegfilgrastim is also the drug they most frequently use for SP (in 84% of patients compared with 15% for filgrastim and 1% for lenograstim).

The oncologists surveyed use a variety of antibiotics in patients who develop FN (Table 4), alone or in combination: piperacillin + tazobactam is the most frequently used antibiotic (used in 34% of patients, on average), followed by amikacin (26%) and ceftazidime (18%).

**Table 4:** Clinical management of FN based on expert opinion

Drug name	**Patients treated among those developing FN (%)**^**a**^	Total daily dose (mg)	Average duration (days)
Piperacillin + tazobactam	34%	16 000	6.1

Amikacin	26%	1 000 to 1 500	4.1

Ceftazidime	18%	2 625 to 4 000	5

Amoxycillin + clavulanic acid	17%	6 000	5.4

Cefepime	14%	6 000 to 16 000	5.8

Filgrastim	13%	0.3	4.5

Fluconazole	8%	200 to 400	6.5

Ciprofloxacin	7%	0.3	5.5

**Procedure name**	Patients treated among those developing FN (%)*	**Average frequency of procedure**	

Chest X-ray	80%	1.2	

Microbiological tests	78%	2.4	

Abdominal echography	7%	1.0	

**Hospitalization by usage of PP/SP**	**Patients hospitalized among those developing FN (%)**	**Average [range] LOS (days)**	

No G-CSF prophylaxis	48%	4.2 [3-7]	

PP	19%	1.5 [1-2]	

SP	20%	3.3 [3-7]	

Only resources with frequencies >5% are reported. The table is based on oncologists' answers. The participating hematologists reported similar findings (results not shown).

LOS: length of stay

^a^Obtained by multiplying the percentage of experts mentioning the drug/test by the average proportion of patients receiving this drug/test as estimated by these experts.

The medical procedures most frequently performed by oncologists in cases of FN are chest X-ray (in 80% of patients, on average), microbiological tests (78%) and echography of the abdomen (7%).

Finally, oncologists estimated the hospitalization rate due to FN at 48% in patients not receiving any type of G-CSF prophylaxis (average length of stay 4.2 days) compared with 19% in patients receiving PP (1.5 days) and 20% in patients receiving SP (3.3 days) (Table 4).

The clinical management of FN reported by hematologists for patients with NHL was overall similar to that reported by oncologists for patients with breast cancer. On average, the hematologists gave however a slightly longer length of hospital stay for FN in NHL patients not receiving G-CSF prophylaxis (5.6 days).

## Discussion

This survey was initiated to collect recent Belgian data on the use of chemotherapy regimens known to be associated with a moderate or high risk of FN in breast cancer and aggressive NHL, and to capture data on the occurrence and management of FN with these regimens in current clinical practice.

The main survey interest is to provide new and usable insights into the use of moderate or high risk regimens in breast cancer and NHL, as well as on the incidence and management of FN in these patient groups in a Belgian daily practice setting.

**Figure 1:**
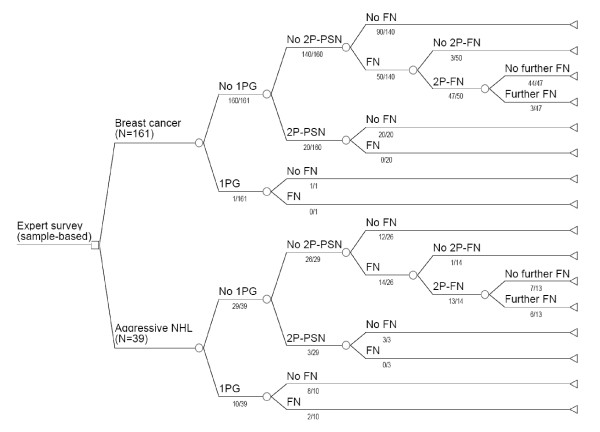
**Tree-like representation of sample-based survey results in breast cancer and NHL**. For each indication, the total number of patients by type of G-CSF prophylaxis received and occurrence of FN/PSN is given under the corresponding branch

A summary of the survey results is presented in Figure 1. The first observation to be made is that the overall incidence of FN with the regimens investigated was higher than that which would be expected from the literature, particularly in cycles not protected by G-CSF prophylaxis (31% of breast cancer patients and 48% of NHL patients had FN in unprotected cycles). Furthermore, these results appear to confirm, in a real-life setting, the efficacy of secondary G-CSF prophylaxis in limiting the occurrence of further FN and PSN, as it was demonstrated in clinical trials [8]. We observed a clear trend for patients with NHL to be at higher risk of neutropenic complications than their breast cancer counterparts (very probably due to more intensive chemotherapy regimens).

In terms of the consequences of FN, 40% of breast cancer patients who developed FN experienced some chemotherapy dose reductions, dropped or delayed cycles, whereas 38% of patients with NHL who developed FN experienced delays in cycles.

The observations from our study can be compared with some results of recently published European studies in breast and NHL patients.

A prospective observational study investigated FN-related endpoints such as occurrence of neutropenic events, dose/delays issues, G-CSF protective effect and FN treatment burden in breast cancer and lymphoma patients in Europe (INC-EU study) [5].

Despite targeting similar endpoints, differences in patient selection make comparison of the results difficult. Indeed, all chemotherapy regimens used were eligible, whereas in our study only regimens categorized as having a moderate or high risk of FN were targeted. As a consequence, a lower rate of neutropenic events was observed in the INC-EU study as compared to the results reported in our study: FN occurred respectively in 6% vs 31% of breast cancer patients and in 22% vs 41% of NHL patients. The use of primary prophylaxis was observed in 9% vs. only 1 patient (<1% of our sample) in breast cancer and in 28% vs 26% of patients in NHL patients. In addition, a protective effect of primary G-CSF prophylaxis regarding cycle 1 FN occurrence and relative-dose intensity was demonstrated via statistical modeling [18].

Also, significantly more dose delays and reductions occurred in patients with FN (breast cancer: 42% with delays, 39% with reduced dose; NHL: 53% and 40%) compared to patients without neutropenic complication, and these limitations occurred in proportions that are in line with our assessment (40% of patients with FN experiencing any type of impairment). Our study however failed to collect dose-limitation data for the patients without FN (see limitations due to survey design below).

The second study is a randomized controlled trial by the Dutch/Belgian HOVON group, conducted in elderly patients aged 65 and above with aggressive NHL, being randomly assigned to receive either CHOP or CHOP+G-CSF [22]. Although this patient group differs significantly from our sample of younger patients (<65) treated with a variety of intense regimens, the reported incidence of FN in this HOVON study is the same as what we observed in our study, namely 41% of patients developing FN (158 out of 389 patients in the HOVON study and 16 out of 39 patients in our study). Despite an only modest difference in FN rates between both arms (37% in CHOP vs. 45% in CHOP+G-CSF), patients with upfront G-CSF prophylaxis received a significantly higher median relative dose intensity of cyclophosphamide (+2.4%) and doxorubicin (+2.1%), and experienced significantly fewer cumulative days with antibiotics (median 0 vs. 6 days).

The latter outcome regarding medical resource use with FN was reflected in our expert opinion survey on the burden of FN treatment (intravenous antibiotics were the most frequently prescribed medication in case of FN). In addition, both oncologists and hematologists mentioned potential benefits of prophylaxis on hospitalizations frequency and duration: they considered both the number of hospitalizations and the length of hospitalization in patients who developed FN to be halved by G-CSF prophylaxis compared with no prophylaxis. These potential reductions in length of hospitalization and hospitalization rates can have a positive impact on patient's quality of life and on treatment cost [23,24].

Another notable finding of our study is that dose-dense or TAC regimens were used in none of the patients with breast cancer and in fewer than 30% of patients with NHL. A possible explanation for this finding is that these highly myelotoxic regimens require, for safe use, upfront prophylaxis against FN, but at the time the survey was carried out primary prophylaxis was not covered by Belgian health insurance agencies for patients aged <65 years.

The main limitations of our study were linked to its design. Participants had to report patient data themselves and therefore the numbers of selected patients and the list of targeted variables were both limited. In addition, some centers easily reached the maximum sample size permitted by the protocol and reported on only a random selection of their eligible patients, whereas other centers could include all eligible patients. As a consequence, a certain selection bias cannot be excluded.

One consequence of this is that extrapolation of our results, especially with the small NHL sample, to a general population should be attempted with caution (e.g. by checking the repartition of population by disease stage). The variability in incidence of neutropenic events or proportion of patients receiving prophylaxis in the NHL sample is also higher than in the breast cancer sample, which could hinder comparison.

In addition, the use of antibiotics reported in the survey could not be investigated sufficiently in depth in order to adjust for FN/PSN incidence following antibiotic prophylaxis, which is known to reduce the risk of neutropenic events. However, antibiotic prophylaxis is not recommended by EORTC guidelines because it can potentially lead to the emergence of resistance [25].

Finally, a number of queries had to be dealt with *a posteriori *to clarify uncertainties regarding the definitions of prophylaxis or the timing of FN occurrence.

Despite the above-mentioned limitations, the outcomes of this study provided real-life data on incidence and consequences of FN in these patient groups, and were subsequently submitted to the Belgian reimbursement commission as part of a request to extend the coverage of primary prophylaxis with pegfilgrastim. As a result, a reimbursement extension for a subgroup of high-risk patients with breast cancer and NHL aged <65 years was granted in September 2009, thereby improving access to FN prevention with G-CSF for part of the population targeted by this survey.

## Conclusions

The collection of real-life data allowed the identification of a gap between clinical practice and current recommendations for G-CSF use according to international guidelines. Our results show that, considering the observed consequences of FN on chemotherapy delivery (dose adjustments, delays, dropped cycles), and the known associated negative impact of such consequences on the efficacy of treatment, patients with breast cancer and NHL who are to receive moderate or high risk chemotherapy regimens may benefit from better access to primary prophylaxis with G-CSF.

## List of abbreviations used

AC-T: anthracyclines, cyclophosphamide followed by docetaxel; ACVBP: doxorubicin, cyclophosphamide, vindesine, bleomycin, prednisone; ASCO: American society of clinical oncology; BVAM: carmustine, vincristine, cytarabine, methotrexate; CHOP-21: cyclophosphamide, doxorubicin, vincristine, prednisone every 21 days; EORTC: European organization for research and treatment for cancer; ESHAP: etoposide, methylprednisolone, cytarabine, cisplatin; ESMO: European society for medical oncology; FEC: 5-fluorouracil/cyclophosphamide/epirubicin; FEC-T: 5-fluorouracil/cyclophosphamide/epirubicin followed by docetaxel; FN: febrile neutropenia; G-CSF: granulocyte-colony stimulating factor; HR: hazard ratio; Hyper CVAD: cyclophosphamide, doxorubicin, vincristine, dexamethasone, methotrexate, cytarabine; IPI: international prognostic index; NCCN: national comprehensive cancer network; NHL: non-Hodgkin's lymphoma; PP: primary G-CSF prophylaxis; PSN: prolonged severe neutropenia; R: rituximab; R-ACVBP-14: rituximab with doxorubicin, cyclophosphamide, vindesine, bleomycin, prednisone for 14 days; R-CHOP-14: rituximab with cyclophosphamide, doxorubicin, vincristine, prednisone every 14 days; R-CHOP-21: rituximab with cyclophosphamide, doxorubicin, vincristine, prednisone every 21 days; R-COPADEM: rituximab with cyclophosphamide, vincristine, prednisone, doxorubicin, methotrexate; R-DHAP: rituximab with cisplatin, cytarabine, dexamethasone; R-GEMOX: rituximab with gemcitabine, oxaliplatin, prednisone; R-ICE: rituximab with etoposide, carboplatin, ifosfamide; RR: relative risk; SP: secondary G-CSF prophylaxis (any type); SP-FN: secondary G-CSF prophylaxis after febrile neutropenia; SP-PSN: secondary G-CSF prophylaxis after prolonged severe neutropenia; TAC: docetaxel, doxorubicin, cyclophosphamide.

## Competing interests

This study is supported by Amgen NV. Amgen provided financial support for English language editing. Laetitia Gerlier, Mark Lamotte and Luc Somers are consultants to Amgen NV.

## Authors' contributions

LG was the main analyst and wrote the paper; she takes primary responsibility for the paper. ML coordinated the research, and did the quality review with LS. Drs Awada, Bries, Bosly, Cocquyt, Focan, Henry, Lalami, Machiels, Mebis, Straetmans, Verhoeven participated in the expert survey and critically appraised the paper. All authors read and approved the final manuscript.

## Pre-publication history

The pre-publication history for this paper can be accessed here:

http://www.biomedcentral.com/1471-2407/10/642/prepub
